# A Short-Term Advantage for Syngamy in the Origin of Eukaryotic Sex: Effects of Cell Fusion on Cell Cycle Duration and Other Effects Related to the Duration of the Cell Cycle—Relationship between Cell Growth Curve and the Optimal Size of the Species, and Circadian Cell Cycle in Photosynthetic Unicellular Organisms

**DOI:** 10.1155/2012/746825

**Published:** 2012-05-14

**Authors:** J. M. Mancebo Quintana, S. Mancebo Quintana

**Affiliations:** ^1^C/J. A. Moreno Fuentes 21, Ávila, 05400 Arenas de San Pedro, Spain; ^2^C/Pilar de Zaragoza 78 4 A, 28028 Madrid, Spain

## Abstract

The origin of sex is becoming a vexatious issue for Evolutionary Biology. Numerous hypotheses have been proposed, based on the genetic effects of sex, on trophic effects or on the formation of cysts and syncytia. Our approach addresses the change in cell cycle duration which would cause cell fusion. Several results are obtained through graphical and mathematical analysis and computer simulations. (1) In poor environments, cell fusion would be an advantageous strategy, as fusion between cells of different size shortens the cycle of the smaller cell (relative to the asexual cycle), and the majority of mergers would occur between cells of different sizes. (2) The easiest-to-evolve regulation of cell proliferation (sexual/asexual) would be by modifying the checkpoints of the cell cycle. (3) A regulation of this kind would have required the existence of the G2 phase, and sex could thus be the cause of the appearance of this phase. Regarding cell cycle, (4) the exponential curve is the only cell growth curve that has no effect on the optimal cell size in unicellular species; (5) the existence of a plateau with no growth at the end of the cell cycle explains the circadian cell cycle observed in unicellular algae.

## 1. Introduction

### 1.1. Theories about the Origin of Sex, Syngamy, and Meiosis

Sex, as a reproduction method, involves two main processes: meiosis—gamete generation or return to a haploid cell state—and fusion of gametes or haploid cells [1]. These two processes are present throughout the whole of the Eukarya domain, indicating that they both appeared simultaneously with the first eukaryotic cells. However, it is unknown why sex appeared and why it is still being used today. Darwin suggested that the reason for sex was to provide “hybrid vigor” [2]. The first to doubt the evolutionary advantage of sex, 30 years later, was Weismann, who gave his own explanation: it increases genetic variability, upon which selection can act [3]. In the 1930s, with the birth of population genetics and Neo-Darwinism, Fisher [4] and Muller [5] reformulated Weismann's argument to incorporate the concept of gene. Sex is explained by intrinsic genetic recombination of sex, which brought together beneficial mutations in a single individual. 

In the 1970s, Maynard Smith [6, 7] and Williams [8] turned this situation around. Their argument is that for a female, a sexual strategy has a twofold cost compared to the asexual strategy, due to the production of males and the lower likelihood of a gene passing to her offspring. However, this problem—the maintenance of sex—is different from its origin, since we assume that the first eukaryotes did not have two separate sexes, and all species were probably isogamous. Even so, in the origin of sex there are two major hurdles to overcome: first, the need for a gamete to find a partner with whom to merge, which must presumably incur a cost in time and energy; and second, the long and complex process of meiosis (the “cost of meiosis” [9, 10]) needed to return to the haploid state. The evolutionary steps from panmixis (all individuals are potential partners) to only two sexes (or two types of gametes) and from isogamy to anisogamy, both closely related, are two additional problems, halfway between “origin” and “maintenance”, and have been the subject of numerous studies [11–18]. For theories about the maintenance of sex, see Bell [19].

Regarding the origin of syngamy, Cavalier-Smith has proposed the following sequence of evolutionary stages: first, a common ancestor of Eukaryotes and Archaebacteria, similar to the current Gram-positive bacteria, surrounded by a cell membrane and a peptidoglican wall [20–22]. Second, the necessity to adapt to a hot acid environment led the common ancestor to lose the peptidoglican wall [21, 23]. Third, those first eukaryotes were therefore characterized by a soft cell that made it easy to evolve phagotrophy. Fourth, a slight modification in the mechanism of phagotrophy then led to syngamy [1, 24–27]. With a picture like this, syngamy appears as almost an inevitable step: Cavalier-Smith goes on to say that “cell fusion is mechanistically easy to evolve and has probably done so on numerous occasions” [25, page 343], and Kondrashov refers to it as “an easy event” [28]. But the fact that the merger *could* occur does not necessarily mean that it actually would. An early idea was the nutrition theory [29]: doubling a protozoan cell's food storage reserves may greatly increase survival in the case of starvation. More recently, Cavalier-Smith proposed that the reason for this is the formation of syncytia and cysts [25, page 342], and trophic causes [27, page 46]. However, Margulis and Sagan have proposed that the origin of syngamy might lie in acts of aborted cannibalism which end in fusion [30, page 97]. 

After syngamy, the cell fusion diploid state is reached, and many theories have thus focused on the advantages of diploidy [31]: protection against somatic mutations [32], protection against inherited mutations [33], the need to repair genetic damage [24, 34–37] (revised in [38]), and increased rates of beneficial mutations [39]. Some hypotheses have, however, described advantages to both haploidy and diploidy depending on the circumstances: *r*-selection favoring small-celled haploids and *k*-selection favoring large-celled diploids [40], selection upon gametic and vegetative cells [19, 41], response to nutrient availability [42], adaptations to different environments [43], or genetic load arguments (via the “asexual ploidy cycle”) [44, 45].

On the origin of meiosis there is a vast literature. The origin of meiosis occurred early in eukaryotic evolution [46], and it should be noted that it was a comparatively simple evolutionary step, as many recombination enzymes already existed and were involved in other processes such as replication, transcription, and repair [47]. In fact, the core replication machinery appears to be the same for both mitosis and meiosis in fission and budding yeasts [48–50]. Some authors suggest a gradual origin of meiosis with all steps favored by natural selection [1, 51–53]. Maynard Smith and Szathmáry [1] proposed an origin of sex, defined as syngamy and meiosis, through successive evolutionary steps: first a haploid-diploid cycle, with endomitosis and one-step meiosis, followed by a cycle with syngamy and one-step meiosis, and finally a cycle with syngamy and two-step meiosis. However, two-step meiosis may have originated by a single mutation that delayed sister centromere splitting and therefore made it possible for meiosis II to occur without a preceding DNA replication [51].

The first and simplest explanation for the origin of meiosis is that it evolved as a mechanism to restore the haploid state [45, 51]. It may also have evolved as a cell cycle repair mechanism to correct accidental polyploidy (caused by failures in mitosis or an accidental nuclear fusion) [51]. Cleveland, from original observations on primitive flagellates, proposed that the haploid-diploid cycle may have started with a spontaneous diploidization by endomitosis, that is, without syngamy [54]. In this case, alternation of phases existed before the evolution of sexual recombination (but see [47, page 138]).

An effect inherent in meiosis is genetic recombination, so some theories have been based on its benefits. The first hypothesis was that of the aforementioned Weismann (it increases genetic variability) [3]. This has been recently reformulated and revised by Burt [55] and Otto and Lenormand [56]. Bagnoli and Guardiani have proposed a “microscopic model,” which “provides a high velocity of movement in the phenotypic space” [57]. Cavalier-Smith, however, suggests that the recombination caused by sex is an incidental consequence of crossing over, rather than the main selective advantage for the origin of sex [25].

The origin of sex has generated a vast literature, to which can be added two further proposals: as a vaccination method [58], and originated by selfish genetic elements such as virus and plasmids [59]. As we have seen, most works are based on the genetic advantages of sex and only a few in “other causes” (trophic or survival in adverse periods). But this does not mean that all the authors base their proposals on a single type of cause. Among genetic studies, the following passage is meaningful: “cell fusion, in fact, doubling the cell food storage greatly increases the survival probability”; but then “even if syngamy originally evolved for trophic reasons, on the long run, sex fixed in populations due to the several advantages of gene mixing” [57, page 491]. And among the works professing “other causes,” is this contribution by Cavalier-Smith: “the prime role (of syngamy) was twofold: to make zygotes larger and to increase their survival rate by being able to store more solid food reserves; to provide genetic redundancy in the dormant cysts so that accidental damage […] could be repaired by homologous recombination among genomes” [27, page 46].

This paper proposes a new immediate short-term advantage for cell fusion (syngamy): fusion of two haploid cells of different sizes during their growth phase can reduce the cell cycle duration of one of them, thereby increasing the duration of the other. This sexual strategy can spread through an entire population for the shorter cycle of sexual cells and could be the first immediate advantage of sex with a reproductive function. We show how this shorter sexual cycle only occurs in poor environments and explains the relation between sexual reproduction and poor environments, which has been roughly explained by previous environmental theories (the advantage of recombination in changing environments). Our hypothesis relies on cell growth speed, the cell cycle profile and its relations with cycle phases, and cell regulation, so the state of the art of these issues has been researched. It should be noted that all the studies on these issues analyze present species, and it is therefore possible that the cell cycle may have been different when eukaryotes first appeared. As there are no theories for the evolutionary history of cell cycles—although biological cycles have been amply studied—we must assume the simplest premise, that is, cell growth, the cell cycle profile and its regulation in the first eukaryotes, were similar to today. 

### 1.2. Cell Growth Speed

The studies on this issue are somewhat contradictory [60, 61]. In 1998, two scientific articles published in the same journal (Microbiology) studying the adjustment of the length of growth in yeast to an exponential curve, reached the opposite results [62, 63]. Cooper [64] revised various works and proposed that the universal pattern of cell growth during the cell cycle is exponential, in opposition to the linear or bilinear growths defended by other authors. The difference between exponential and linear growth is that with exponential growth the absolute increase in cell mass increases during the division cycle, but with linear growth the absolute increase in cell mass is constant during the division cycle. This exponential growth has been verified in budding yeast (*Saccharomyces cerevisiae*) [65–67], fission yeast (*Schizosaccharomyces pombe*) [68, 69], and in single-cell green algae such as *Chlamydomonas eugametos*, based on the authors' own measurements over microphotographs by Zachleder and Van Den Ende [70]. Baumgärtner and Tolić-Nørrelykke showed an excellent growth curve fit, with *r* ^2^ > 0.99, although fitting a bilinear curve was still major [68]; this result has been confirmed by other authors [71]. In any case, whether the curve is bilinear or exponential, the larger the cell size, the higher the growth rate. Godin et al., using a suspended microchannel resonator (SMR), which measures mass with femtogram precision, found that for individual cells of *Bacillus subtilis, Escherichia coli, Saccharomyces cerevisiae*, and mouse lymphoblasts, heavier (bigger) cells grow faster than lighter (smaller) cells [72].

Assuming that the exponential growth (or similar) was valid for the first eukaryotes, the hypothesis of Cavalier-Smith [73] and Maynard Smith and Szathmáry [1] that haploid cells—being smaller—would have a higher growth rate than diploid cells, is probably false. Their argument is as follows: haploid cells are often smaller than diploid cells [40, 74] and thus have a higher surface area to volume ratio. As the ability to transport nutrients across the cell membrane depends on surface area, this increased ratio may lead to improved growth rate or survival, especially under nutrient limited conditions (the “nutrient limitation” hypothesis). However, recent experiments using an isogenic series of haploid, diploid, and tetraploid yeast cells (*S. cerevisiae*) do not support the nutrient limitation hypothesis [75].

### 1.3. Cell Cycle Regulation

Various classic studies [76–80], and some more recent ones [60, 66, 81–85] show the presence of critical sizes throughout the cell cycle. In eukaryotes, these critical sizes mark the G1 to S transition (called the G1/S control point or checkpoint) and/or the G2 to M transition (G2/M checkpoint). As these checkpoints are the same among unrelated species, it is very likely that these control mechanisms are common to all organisms (bacteria, protists, yeasts, algae, etc.) [66, 83]. It should be noted that the way in which cells measure their size is still unknown, although probable candidates include number of ribosomes, protein quantity, and cell volume [83, 86]. Photosynthetic organisms such as *Chlamydomonas* are also likely to have a double control based on time and cell size [87, 88].

### 1.4. Cell Cycle Profile and Phases

**Figure 1:**
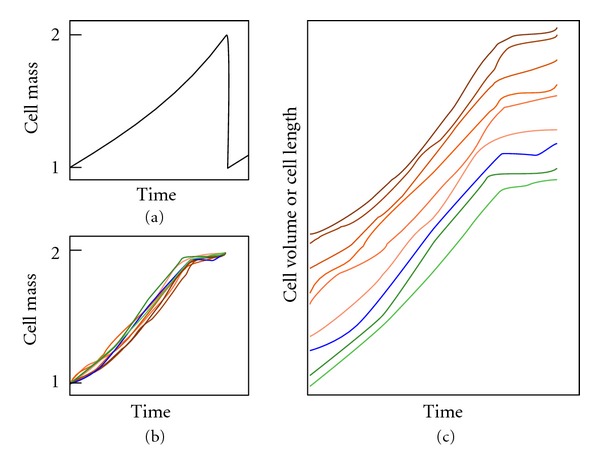
Eukaryotic cell cycle profiles in modeling studies (a) and studies measuring the individual growth of single cells in fission yeast (b) and (c). The first assumes a triangular profile; while in the second, a final plateau always appears with no growth or very limited growth. The profiles in (b) and (c) are taken from Baumgärtner and Tolić-Nørrelykke [68, page 4337] (six profiles with red tones measuring the parameter of length), from Neumann and Nurse [69, page 596] (one blue profile: cell volume) and from Buchwald and Sveiczer [71, page 5 and 6] (two green profiles: length). The horizontal and vertical scales have been modified to match the beginning and end of every cycle, to better show the presence of the plateau. In figure (c) they have also been arranged vertically. Cells of fission yeast are cylindrical, with a constant diameter, and grow by extending at the ends; therefore, cell length is a good measure of the size (volume and mass) of these cells [101].

**Figure 2:**
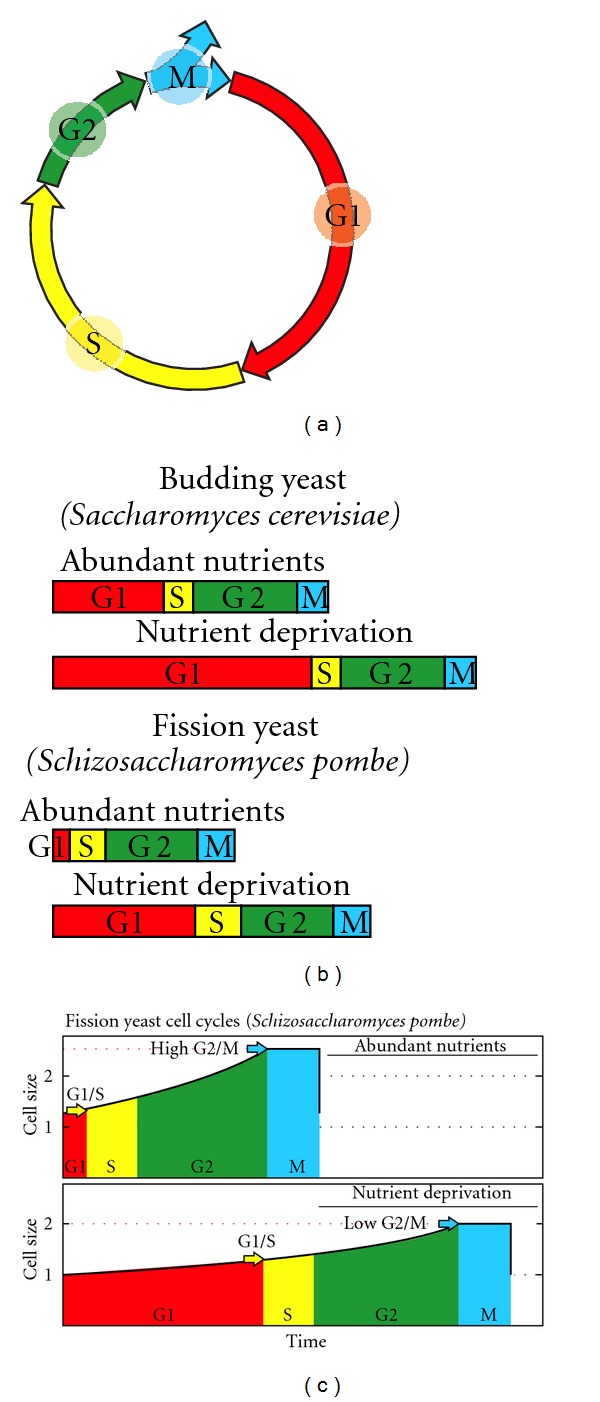
Cell cycle, cycle graph as shown in regulation studies (a), (b) and proposed cycle profile graph (c). (a) Phases of the present eukaryotic cell cycle. (b) Common cycle graph as shown in regulation studies. The duration of S, G2, and M tends to be constant in each species, although the duration of G1 varies depending on the richness of the environment. (c) Proposed graph, including cycle profile and critical sizes of checkpoints.

**Figure 3:**
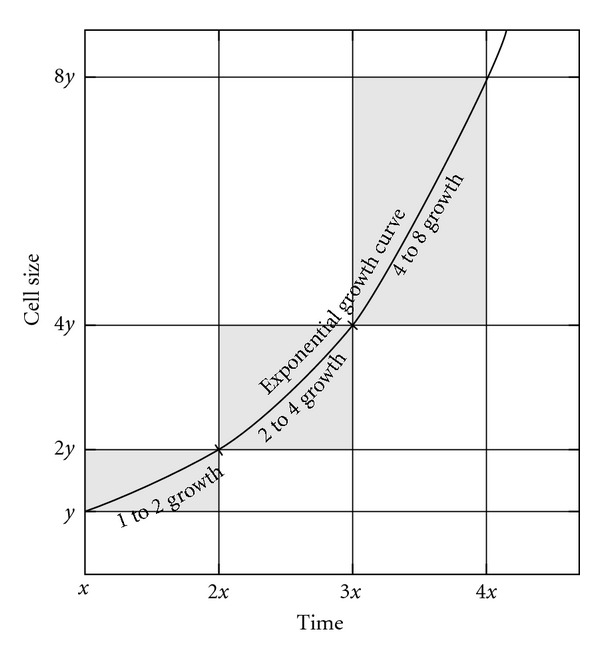
Exponential growth curve. This is the only curve in which duplication time is independent of cell size. Compare with Figure 1(d) in [72].

There is no consensus as to the existence of a plateau with no growth or with diminished growth at the end of the cell cycle. According to Mitchison, the commonest pattern for cell growth is one of continuous increase through the cycle [89, page 147]. Similarly, studies on cell cycle modeling assume that cells grow throughout the whole cell cycle, including the division phase, thus showing a triangular time-size profile, without a plateau (Figure 1(a)) [90–98]. Nevertheless, when present cell growth is measured and analyzed, there is a reduction or even a lack of growth at the end of the cell cycle, that is, a final plateau in the time-size profile (Figure 1(b)), as observed in fission yeast (*Schizosaccharomyces pombe*) [68, 69, 71, 99–101], amoebas [102], *Physarum* [103], *Paramecium* [104], and *Tetrahymena *[105]. Different authors refer to it as *plateau*, *constant volume (or length) phase*, or *constant size period*. We examined empirical studies that show this plateau, and found that growth stops in some [69, page 596, Figure 3(B)] [71], and is slow in others [68, page 4337, Figures 1(d) and 2]. Regarding the percentage of cell cycle, Baumgärtner and Tolić-Nørrelykke [68, page 4339, Table 1] analyzed this in fission yeast and their results showed that the plateau represents 14.5 ± 3.9 percent of the generation time at 25°C, 18.5 ± 1.4 percent at 28°C, and 22.6 ± 6.8 percent at 32°C. The plateau, as expected, corresponds to nuclear division and cytokinesis [101, page 374], [68, page 4338], and [71, page 9].

We have therefore outlined this relationship using the current knowledge of the cell cycle and its regulation: (1) the present cycle has four phases: G1 (from gap), S (DNA synthesis), G2, and M (mitosis) (Figure 2(a)). The M phase comprises two major events: nuclear division, or mitosis, during which the copied chromosomes are distributed into a pair of daughter nuclei; and cytoplasmic division, or cytokinesis, when the cell itself divides in two. (2) There is a lack of growth during division, M phase, as stated above. (3) The duration of S, G2, and M tend to be constant in each species, although the duration of G1 varies depending on the richness of the environment [66, 106–108] (Figure 2(b)). In fission yeast growing at different temperatures, the duration of the plateau is constant [68, page 4340]. (4) There are two checkpoints—related to cell size—in G1/S and G2/M [66]. The result is a trapezoid profile (Figure 2(c)) in which the duration of G1 varies according to the environmental richness, showing a final phase with no growth, which corresponds to the division phase, and showing a constant adult cell size, self-controlled by G2/M. The profile of the cycle also depends on the size distribution of the daughter cells. We assume this distribution is the one most commonly found in present prokaryotes and eukaryotes, that is, a symmetrical division. 

### 1.5. Ploidy and Cell Size

Cell size is related to ploidy; in each species, polyploid cells are larger than diploid cells, and these are in turn larger than haploid ones. This relation can be observed in many eukaryotes, from yeasts to mice [97, 109–113]. In yeasts, triploid cells are medium-sized compared to tetraploid and haploid cells [114]. In fission yeast, diploid cell volume is 1.5–1.8 times that of haploid cells [115]. In budding yeast, the critical size at Start (G1/S checkpoint) increases in proportion to cell ploidy level and nutrient status [116]. The reason for this relation lies in the mechanism of regulation by checkpoints. This mechanism is based on the production or inhibition of cyclin, whose quantity depends on ploidy level [114].

### 1.6. Duration of the Ancestral Meiosis

Regarding the duration of meiosis, ancestral meiosis may have been short if its main function was not genetic repair or genetic recombination; in meiosis, bivalent formation is not always necessary to achieve segregation, although it is the most common means in species today [117]. Moreover, in polyploid plants, there is usually a negative correlation so that high ploidy levels are connected to short-duration meiosis, despite the higher DNA content [118, 119].

## 2. Materials and Methods

The software (CELLSIMULATOR) was programmed to simulate the biological evolution of a single-cell population and to test the hypothesis regarding the advantages of syngamy. It was programmed using visual basic for applications and works in an Excel spreadsheet (additional file: *cellsimulator26.xlsm*).

In this simulator, the population is a matrix or table of organisms with some parameters. Cells do not occupy a position in space, so no spatial effect can be analyzed (cell dispersion, nutrient diffusion, cell encounters, etc.). Each organism has the following.

- 5 main parameters: identification number, age, cycle phase, size, and ploidy level.
- 1 or more genomes, depending on ploidy. Each genome has 4 chromosomes.
- The first chromosome has
  1. 3 main genes: mitosis symmetry, G1/S critical size, and G2/M critical size,
  2. 1 gene that controls sexual strategy: asexual, sexual, or shielded,
  3. 3 genes when sexual: meiosis I symmetry, meiosis II symmetry, and phenotypic control of critical sizes when diploid.
- The other three chromosomes have
  1. 2 genes: growth capacity and survival capacity.
- Every gene has a complementary gene to control its mutability.

Some population parameters are controlled by the user: total quantity of nutrients—either constant or variable; maximum absorption capacity of each cell; growth curve; nonabsorbent volume (e.g., the nucleus); biological efficiency of cell size—either a lognormal curve or a min-max range; random survival rate; symmetry random segregation; symmetry mutability; checkpoint mutability; other gene mutability; S phase duration when haploid; S phase duration when diploid; division duration; and fusion rate.

Briefly, it simulates a dynamic single-cell population. In each time unit, cells grow depending on available resources (a function of population size) and growth genes (a function of its volume or area). When a cell reaches the G1/S checkpoint (genetically controlled), the S phase begins and the genome duplicates (mutations occurring in the genome copied with a given rate). Mutation varies gene value; this variation is given by the complementary mutation control gene. This control gene also mutates; the quantity of mutation is modified as time goes by. The cell continues growing in G2. When the G2/M checkpoint is reached, division begins, chromosomes segregate between daughter cells, and cell volume splits the function of symmetry gene. The durations of S and M phases are also parameters.

At the same time, cells may die during each time unit. Death comes randomly with a probability that varies with a range or a lognormal curve. Survival genes modify this probability. Sexual cells fuse other cells with a probability that varies with population density. The phenotypic expression of diploid cells is the average of the gene values, but for growth and survival genes it is the maximum, simulating a compensation of deleterious mutations, that is, hybrid vigor.

To analyze population evolution, the simulator records a history with some parameter statistics and shows the results in graphs. During the simulation, the user can take certain actions such as adding an asexual cell, adding a sexual cell, mutating a cell from asexual to sexual, mutating a cell from asexual to shielded, recording fusions, tracking lineages, and so forth Cells can be identified with a number that is inherited, so that lineages can be tracked in an asexual population.

## 3. Results

### 3.1. Growth Curves and Species Optimum Size

**Figure 4:**
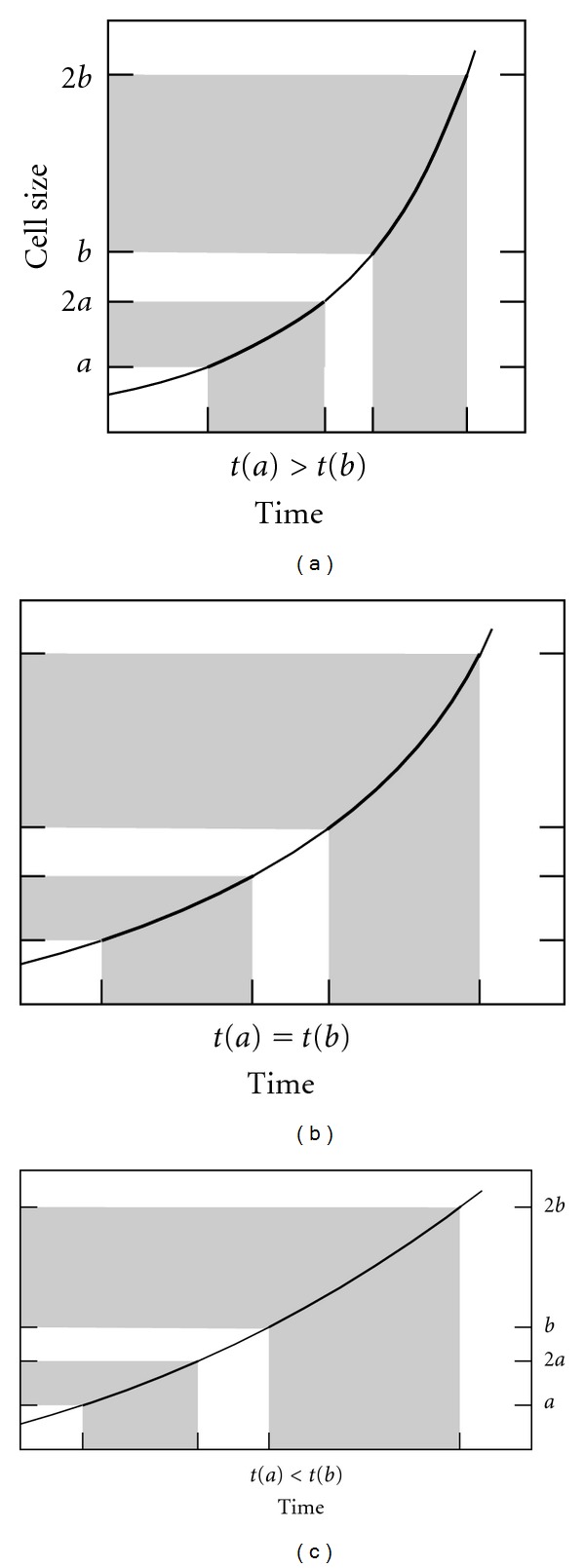
Size/time graphs showing duplication time for different growth curves. (a) Overexponential. (b) Exponential. (c) Subexponential. If the growth rate is subexponential (including linear growth), the smaller the cell, the shorter the duplication time and vice versa. Optimum size will be strongly influenced by these curves. Surprisingly, this fact has not been taken into account, despite its huge importance for single-cell organisms.

For single-cell organisms, an exponential growth rate has a crucial effect on generation time: this is the only rate for which the time needed to duplicate the cell size is independent of the cell size itself (Figure 3). Hence optimum size for a given species is determined by numerous factors, excluding growth rate. If the growth rate is subexponential (including linear growth), the smaller the cell, the shorter the duplication time and vice versa (Figure 4). Optimum size will be strongly influenced by this type of curves. Surprisingly, this fact has not been taken into account, despite its huge importance for single-cell organisms.

Exactly how important is the growth curve in the doubling time? We will calculate this in the case of linear growth, which is a particular subexponential curve:

(1) $$S_{t}=S_{0}+kt,$$

where *S*_*t*_ is the cell size at time *t*, *S*_0_ is the cell size of the new born cell, *k* is the constant growth rate, and *t* is the time.

Resolve *t*:

(2) $$t=\frac{S_{t}-S_{0}}{k},$$

and as *S*_0_ = 1/2 · *S*_*T*_ (being *S*_*T*_ the cell size of the mother cell in division time, *T*),

(3) $$T=\frac{S_{T}}{2k}.$$

This means that there is a direct relationship between the duplication size and the duration of the doubling time: a *j* percent reduction in cell size (initial or final) results in a *j* percent reduction in the length of the cycle.

**Figure 5:**
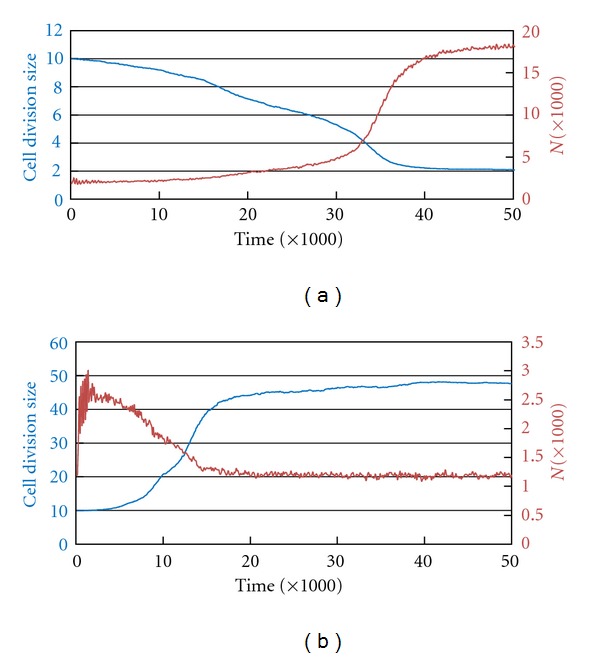
Evolution of the critical size of division (G2/M) in cell populations with subexponential growth ((a), coefficient = 0.5) and overexponential ((b), coefficient = 1.5) in a simulation where CELLSIMULATOR allowable limits are 1 and 50. In both cases, the founder of the population has a critical size for division of 10. The mutation rate of G2/M is 10%. In (a) the critical size for division is stabilized slightly above 2, as bipartition thus generates two daughter cells of the size closest to 1. (b) is stabilized just below 50. (In (a), the average generation is 60, so the simulation includes about 800 generations. In (b), it is 30, so the simulation includes about 1600 generations.)

With our cellular automaton (CELLSIMULATOR) we have found that this effect is in fact real (as real as a computer simulation can be). To do this (Figure 5), we set out to evolve an asexual population whose cells grow following a subexponential or overexponential curve, and we observe variations in the critical division size (on the average of the population). So that the simulations show the effect quickly, we introduce a high mutation rate (10%) in the checkpoint G2/M. If the growth curve is subexponential, the critical size for division develops into the smallest possible size (up to the limit imposed in the simulation), increasing the number of individuals. If the curve is overexponential, the opposite occurs: the size evolves towards the largest possible. Both results confirm our hypothesis.

### 3.2. Final Plateau and Circadian Clock in Unicellular Species

There are numerous examples of microorganisms with 24 h cell division cycles in which DNA replication and cell division occur during the night [120]. One particularly interesting case is that of single-cell green algae (e.g., Chlamydomonadaceae), which grow in daylight, and division—by multiple fission and with no evidence of growth—occurs in darkness [70, 87, 88]. To explain these “circadian cell cycles,” two hypotheses have been proposed. First, that the division occurs at night because the mitosis prevents RNA synthesis, resulting in delayed growth if done during the day [87, page 188]. The second, termed “light to escape”, maintains that circadian clocks evolved early in life, when ultraviolet light could damage the DNA during replication of the genome [121].

**Figure 6:**
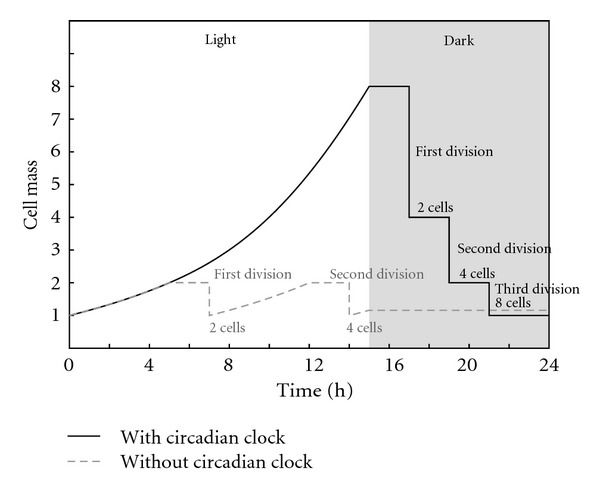
Cell cycle profiles with and without circadian rhythm in unicellular photosynthetic species. The strategy of delaying cell divisions to the period without light makes perfect sense when considering the plateau with no growth at the end of the cycle. The strategy with circadian rhythm and multiple fission would produce 8 cells day and the other only 4.

However, this phenomenon can be clearly explained if we admit the existence of the plateau with no growth at the end of the cell cycle, as there is an obvious advantage in moving the plateau to the nocturnal phase of the day. Thus, cell growth is possible, without interruption, during daylight hours (Figure 6). We found no studies on the cell growth rate in algae showing the existence of the plateau, except the aforementioned work of Craigie and Cavalier-Smith on *Chlamydomonas reinhardtii * [87], which charts numbers 1A and 5 suggest that there is indeed a delay before the first division occurs. Although these authors point out an advantage related to a continuous growth during daylight, they do not conclude the smaller duplication time and hence, a bigger offspring. Instead they argue that “the function of multiple fission is to facilitate the temporal separation of growth and division” [87, page 188].

In unicellular photosynthetic species with binary fission, a link is also expected between the growth phase of the cell cycle and the time of maximum radiation of the day.

### 3.3. Short-Term Advantage for Cell Fusion in Poor Environments

Our hypothesis begins by assuming that the fusion of two haploid eukaryote cells is viable during their growth phase, prior to genomic synthesis (equivalent to modern G1). We assume that the fusions would be viable only in G1 phase because, following Friedman [122], cell cycle activity associated with gamete differentiation and sexual fusion in most eukaryotes is relatively invariant: gametes remain within the G1 phase of the cell cycle through the completion of karyogamy, resulting in a zygote nucleus with 2C DNA content. This basic pattern has been documented (taken from Friedman [122]) in green algae [123–127], brown algae [128–130], red algae [131], yeast [132], ciliates [133–135], and sea urchins [136–138]. Budding yeast cells of each haploid type produce a secreted peptide mating factor which cause cells to arrest in the G1 phase of the cell division cycle, just before the initiation of DNA synthesis [139, 140]. Similarly, most metazoans (from worms to vertebrates) conform to a pattern of fertilization in which mature sperm remain within the G1 phase of the cell cycle through the initiation of cell fusion with an oocyte [141, 142]. Nevertheless, *Arabidopsis thaliana* and other seed plants can be inferred to have G2 karyogamy [122].

The cell fusion mechanism may also have consequences on cell cycle duration if growth stops during fusion. As no evidence was found, we assume growth continues while fusion is taking place.

 The resulting diploid cell would have its cell cycle duration modified through its checkpoints, due to the higher cell ploidy. The occurrence of this *sexual cell cycle* would depend on random cell encounters. In the event that no encounter occurs, the haploid cell would continue its asexual cycle, and would therefore be a fortuitous sex. As these encounters are random, cell ages and cell sizes would also be random. When sex first appeared, there could not have been any true gametes, and thus concepts such as isogamy and anisogamy could not be applied. There are no sex genders, and all cells are potential gametes (panmixis). Then we show how these cell fusions, when occurring between cells of a different size, reduce the duration of the small cell cycle and extend the duration of the large cell cycle. The duration of the small cell cycle can be even shorter than the asexual cycle in poor environments. 

Assuming cell fusion is viable during the G1 phase, we analyze the probable development of the cell cycle after the fusion of two haploid cells. First we examine the case in which both cells are the same size and are right in the middle of G1. The relationship between ploidy and critical division volume has been described in *Saccharomyces cerevisiae* [66, 114] and other studies (see Section 1.5); thus the fused cell, diploid by syngamy, should have larger checkpoints; we will make them exactly two times those of the haploid cell cycle. 

**Figure 7:**
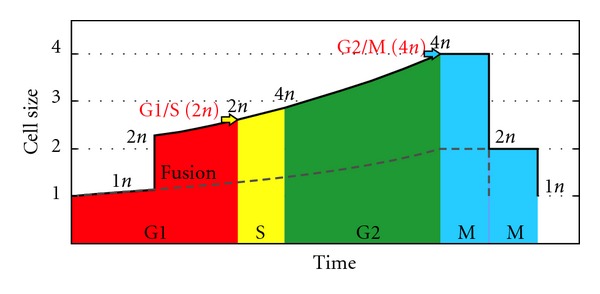
Hypothetical sexual cell cycle involving two equal-size cells. Hypothetical sexual cell cycle involving two equal-size cells in the middle of the G1 phase (black line) compared to asexual (dashed grey line). Both cells are of the same size and are right in the middle of G1. The checkpoints of the cell cycle depend on cell size and ploidy as shown above; thus the fused cell, *diploid by syngamy*, should have larger checkpoints; we will make them exactly two times those of the haploid cell cycle. In this case, the sexual cell cycle is longer than the asexual cycle, due to the second division.

In this case (Figure 7), the new fused cell is still in the G1 phase, as it has yet to reach the G1/S critical size where checkpoints have doubled to the 2n condition. The growth rate is higher than in asexual cells for the larger size. DNA duplication begins once G1/S is reached, and the cell becomes tetraploid (4n). The duration of the S phase may be longer than for haploid cells, although the cell cycle profile would be the same as the cell continues growing; in this case, the G2 phase is shorter but the duration of the S and G2 phases together is the same. It should be noted that the relation between ploidy and S phase duration is not clear [110]. The premeiotic S phase appears to be longer than the pre-mitotic phase; in budding yeast it is 3.5 times longer [143]. This longer S phase would be necessary to establish the interhomolog interactions required for meiotic recombination and faithful segregation of homologous chromosomes [144]. Indeed, the S phase is shorter when recombination is inhibited in budding yeast, suggesting that preparation for recombination is a key process in the premeiotic S phase [145].

Once the synthesis is complete, the cell grows until it reaches the G2/M critical size, which is larger due to its being 4n. If the division mechanism depends on ploidy level—as for some green algae today (*Chlamydomonas eugametos*) [70]—two consecutive divisions would occur, and the offspring would thus return to the original haploid state. In this case, the sexual cell cycle is longer than the asexual cycle, due to the second division. See Appendix A for a mathematical analysis of cycle duration (sexual, Case 1: same size).

We followed the hypothesis which was less favorable, namely that growth stops at the end of the cell cycle. We also consider that the duration of this plateau is constant. Nevertheless, the duration and slope of this plateau have a considerable bearing on the advantages of the sexual cell cycle we describe (see Appendices C and F), pointing to a need for further evidence on this subject.

It should be highlighted that the 4n state is reached *naturally* in this sexual cell cycle, without any changes in the regulation mechanism of the cell cycle. The only unusual fact is that both cell size and ploidy level are higher due to cell fusion.

**Figure 8:**
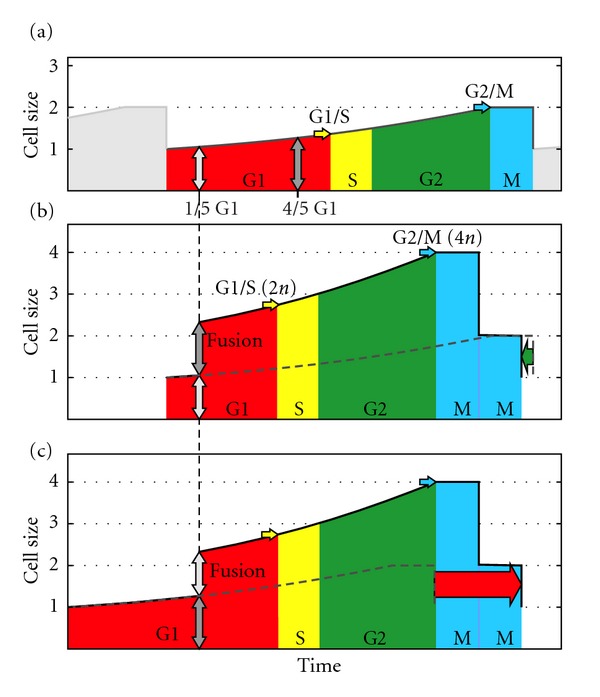
Hypothetical sexual cell cycle involving two different-size cells. (a) Cell sizes at 1/5 and 4/5 of the G1 phase (asexual cell cycle). (b), (c) Small and large cell cycles, respectively. Both sexual cell cycles (black line) are compared to asexual one (dashed grey line). The sexual cycle of the small cell is shorter than the asexual cycle (green arrow). The sexual cycle of the large cell is longer than the asexual cycle (red arrow).

The sexual cell cycle shown—involving two equal-size cells—is unlikely to be usual, as it is fortuitous and dependent on accidental encounters between cells (see Appendix B). We now analyze a second case, the sexual cell cycle when two cells of different ages—hence different sizes—meet and fuse. The younger cell age is 1/5 of G1, and the older cell age is 4/5 of G1 (Figure 8). The duration of the cell cycle *after fusion* is the same for both cells—they are now only one cell—and depends on the total size when fusion occurs. Since the duration of the cell cycle *before fusion* is different for both cells, the total duration of both cell cycles is different. The smaller cell experiences a leap forward in time and it reaches the G1/S checkpoint sooner. Indeed, this time reduction could be enough to compensate for the extra second meiotic division, resulting in a shorter cell cycle than the asexual one. Conversely, the larger cell undergoes a longer cell cycle. A shorter cell cycle confers an immediate advantage. In fact, such a strategy would spread directly through the entire population, making it totally sexual (see Appendix A for a mathematical analysis of cycle duration (sexual, Case 2: different sizes), Appendix D for the influence of sexual G2/M checkpoint, Appendix E for the influence of asexual G1/S checkpoint, and Appendix F for the influence of the plateau duration). 

The advantages of the proposed sexual cell cycle hinge on the preexistence—prior to the discovery of fusion—of the mechanisms responsible for haploid cell return. Otherwise, the cell would remain in a diploid state after cell fusion and there would be no net multiplication; without multiplication, the sexual cycle would have no advantages. 

#### 3.3.1. Testing the Hypothesis with a Cell Automaton

Indeed, could sex have spread so easily? To answer this question we programmed a cell automaton, in which a sexual mutant cell was introduced into an asexual population. Indeed, the results show that sexual strategy spreads fast and easily when the G1 phase is long enough in comparison with cell division. Asexual strategy ultimately becomes extinct. Moreover, simulations showed that the origin of sex was also possible even with the introduction of a cell with a very low probability of fusion.

Using the CELLSIMULATOR, we analyzed two possible types of sex: a yes/no sexuality and a continuous one.

**Figure 9:**
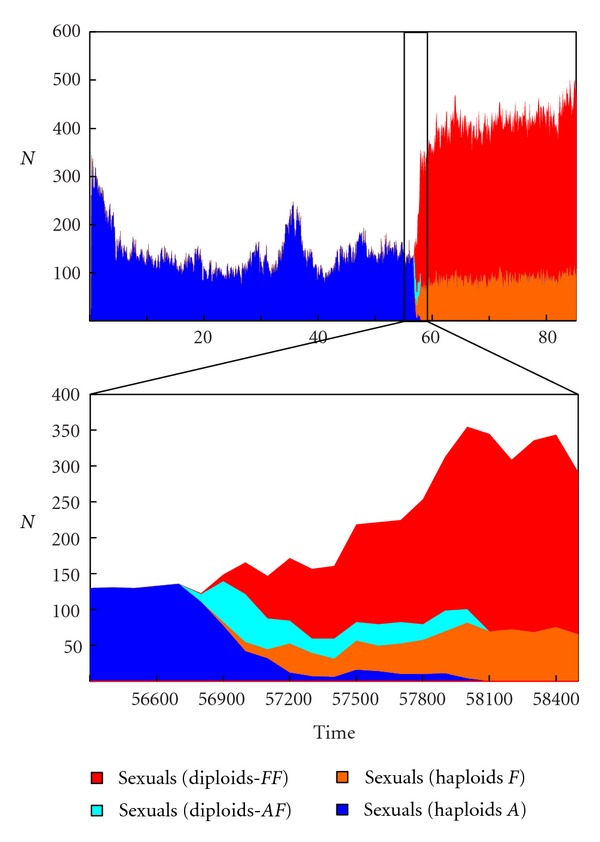
Simulation of a population change from asexual to sexual, with CELLSIMULATOR. Top: complete history. Bottom: zoom to changing period. In *t* = 56700, an asexual cell mutates to sexual; in *t* = 58100, the entire population is sexual. A generation lasts about 12 time units, so substitution occurred in 115 generations. (A: asexual haploids; F: sexual haploids; AF: heterozygous asexual-sexual diploids; FF: homozygous sexual diploids). Population size increases due to the smaller cell size caused by the selective pressure of sexual strategy.

1. In yes/no sexuality simulations, fusion only depends on the probability of encounter, given that at least one of the cells is sexual. The results show that sexual cells can be extinguished when the sexual population is still small, due to random deaths. When the sexual population reaches a certain size and conditions are favorable, the sexual population increases in size and the asexual population becomes extinct. Heterozygous fusions are common (*FA *genotype) when expansion begins, while homozygous fusions predominate in later stages (*FF* genotype). The simulation was terminated when all asexual types were extinct (*A* and *FA* genotypes). In Figure 9, we show an example of sexual change from a single sexual cell. It is often necessary to conduct several trials in order to achieve the goal. Obviously, the more favorable the conditions for sex, the easier for the population to become sexual, although there are only two essential requirements: a long enough G1 phase, plus a short enough plateau. In the next section, we analyze threshold conditions for sex to thrive in an asexual population (relative duration of G1, relative duration of the plateau and probability of fusion).

**Figure 10:**
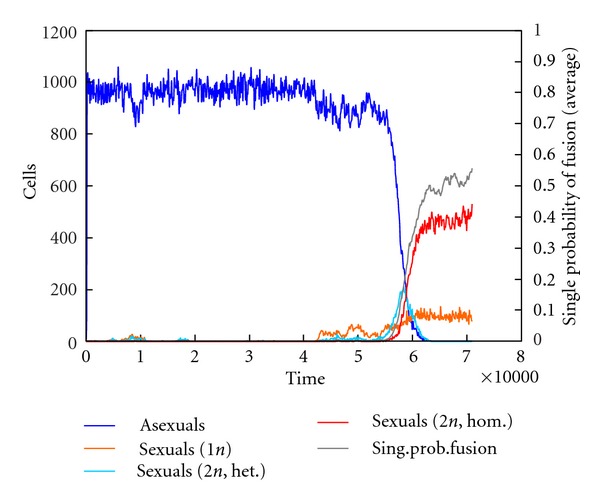
Simulation of a population change from asexual to sexual from one sexual cell with a fusion probability of 2.5%, with CELLSIMULATOR. After various attempts, the sexual strategy displaces the asexual one. The graph shows the number of cells for each genotype. It also shows the evolution of fusion probability, which rises fast during the change and slowly once the population is totally sexual.

**Figure 11:**
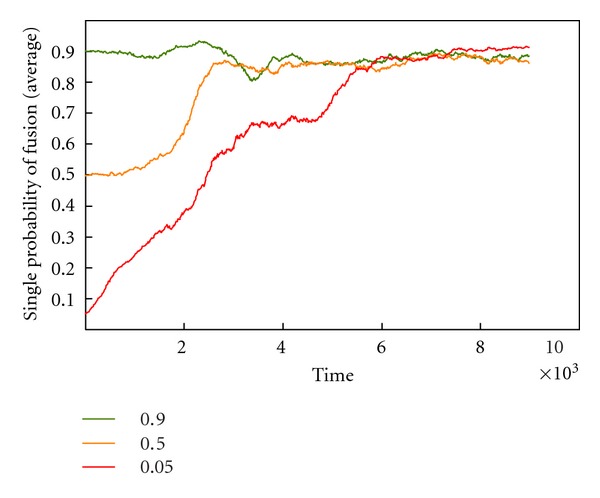
Evolution of fusion probability from a founder with 0.9, 0.5, and 0.05 values, with CELLSIMULATOR. In all cases, the fusion rate tends to reach the maximum value (it is never reached because of the continuous variance created by mutation).

The additional file “simulation-success_of_sexual_strategy” shows a video made with CELLSIMULATOR in which the sexual strategy spreads throughout a previously asexual population.
(2) The software was then updated so that the sexual strategy became continuous. In this case, fusion probability is not given by a constant rate but by the sexual gene, whose value affects the fusion probability (as though the fusion genes were related to the number—or power—of mating factors in the cell membrane). Thus fusion depends on cell encounter probability and cell recognition capacity, given that at least one cell is sexual. Using this type of simulation, it is possible to determine whether sex could have appeared slowly, beginning with a small fusion probability which would change slightly through mutation, and then increase due to natural selection. Regarding the first challenge—the beginning of sex with a low fusion probability, ≤0.1—we observed that this has to occur several times to overcome random cell death, but that ultimately it thrives (Figure 10). Regarding the second challenge—an increase in fusion probability—we verified that cell recognition tends to rise to the maximum allowed value (Figure 11).

#### 3.3.2. Conditions Required for Sex to Thrive

A long series of simulations were performed to obtain the values for certain conditions that would allow sex to thrive:

- relative duration of G1: 20, 40, 60 and 80 (in percentages),
- relative duration of the plateau: 1, 5, 10, 15, 20 and 25 (in percentages),
- fusion probability of sexual cells: 1, 5, 10, 25, 50 and 100 (in percentages).

The total number of simulations was 138 (i.e., all combinations except 80% G1 and 25% plateau due to their impossibility). A macro was programmed to carry out these simulations: it creates a stable asexual population, adding some asexual cells and allowing 5000 cycles to elapse; then it adds one sexual cell and allows 1000 cycles to pass; if all sexual cells become extinct, it repeats the former task; if both sexual and asexual cells are present, it allows 1000 cycles to pass; the process is looped until sex gains or until 105 000 time units is reached. The death rate is calculated to make the asexual cell cycle last an average of 100 cycles. Thus the simulation can include up to 1050 generations, and 100 introductions of one sexual cell can occur.

**Figure 12:**
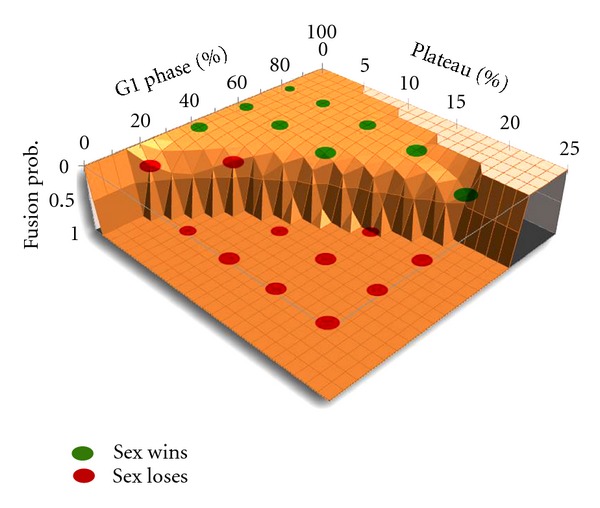
Biological space (combination of factors) where sex is able to displace the asexual strategy. Each point in the surface defines a combination of values for G1 relative duration, plateau relative duration, and fusion probability that allows the origin of sex. This surface is given by a spreadsheet simulation as shown in Appendix C. The red points show the combinations of G1 and plateau where sex did not succeed using CELLSIMULATOR; the green points show where it did succeed. Both results match very precisely. The lighter color surface is an impossible space (combinations of G1 and plateau durations that cannot exist).

The results (Figure 12, red and green circles), which closely fit the theoretical thresholds (Figure 12, 3D graph; see Appendix C), show that sexual strategy is not advantageous if the plateau lasts 20% (or more) of the cell cycle; if the plateau is 10%, the G1 phase must be at least 60% for sex to thrive; and if the plateau is rather short, sex could be a favorable strategy even with a G1 phase as short as 25%. Baumgärtner and Tolić-Nørrelykke [68, page 4339, Table 1] analyzed the plateau in fission yeast and their results were that it represents 14.5 to 22.6 percent of the cycle (depending on temperature).

In the previous explanation of our hypothesis, we assumed there was no variance in the relation between age and size amongst cells, with the aim of making it simpler. However, this assumption is necessary neither in theory nor in practice. Using the CELLSIMULATOR, we tested the introduction of sex in populations showing variance in checkpoints and growth rate, and once again obtained the result that sex is an advantageous strategy.

#### 3.3.3. Asexual Return

Once the population has become sexual, the size range of haploid cells in G1 is reduced, especially when fusions are very likely to occur. Thus, paradoxically, the sexual cycle could become disadvantageous once asexual cells have become extinct. In fact a shield strategy preventing fusions—compulsory-asexual—could be an advantage and return the population to its former asexual condition. However, we consider this to be somewhat improbable, as an effective shield strategy requires a total shield, and the appearance of a total shield in one generation is very unlikely.

The software of CELLSIMULATOR allows the user to test an asexual return by mutating a cell into *shielded-asexual*, preventing fusions. It is thus possible to test the proliferation capacity of a compulsory-asexual lineage competing against a sexual population. Simulations showed that the shielded-asexual strategy thrives fast when a total shield mutation is entered and the cell overcomes random death, thereby bringing sexual strategy to extinction. In the second case, the shield strategy was introduced slowly, slightly diminishing the fusion probability and allowing it to evolve towards a total shield. Despite many attempts, shield strategy never succeeded. It should be noted that neither did the total shielded-asexual strategy succeed when the growth and survival genes were activated, suggesting that hybrid vigor made sexual strategy more efficient.

#### 3.3.4. Short-Term Advantage, but Only in Poor Environments

**Figure 13:**
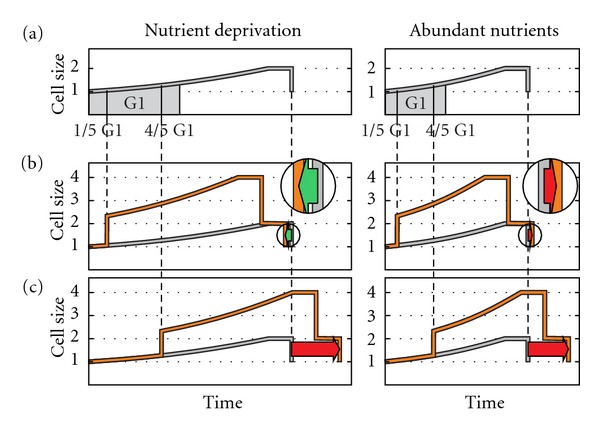
Comparison of asexual (a) and sexual (b), (c) cell cycles in different environmental richness: poor on the left, rich on the right ((b), small cell, and (c), large cell). Cells have the same size before fusion in both environments (the small cell is at 1/5 of G1, and the large cell is at 4/5 of G1), although the sexual cycle of the small cell is only shorter than the asexual cell cycle in poor environments.

We have seen that a fusion between different size cells involves a leap forward in time for the smaller cell; this leap must be enough to compensate for the time taken for the second division in order to make the sexual cycle advantageous. The longer it takes a cell to complete the G1 phase, the more time gained. As the duration of the G1 phase depends on environmental richness, the time gained in poor environments may be shorter than the second division (Figure 13). In present single-cell eukaryotes, meiosis only occurs in poor environments, which means that sexuality is only advantageous in such environments. Hitherto the only explanation given for this fact was environmental: recombination would be useful in changing environments (*If everything is going well, do not change your genetic combination; if things are going badly, recombination and meiosis may be the best option*) [146].

Using the mathematical analysis developed in the Appendix A, we proceed to *calculate* the conditions under which syngamy would be advantageous. In the aforementioned appendix we obtained an equation of the cell cycle duration as a function of the difference of cell sizes (*p*), the rate of cell growth (*k*), and the duration of the plateaus of the two reduction divisions (*T*_*M*_):

(4) $$T=\frac{\ln\left(4\;/\left(1+p\right)\right)}{k}+T_{M1}+T_{M2}.$$

Introducing different values of *k* and *p* in a double entry table, we calculate the exact reduction or enlargement of the small cell cycle. High *p* values would occur whether the G1 phase is long and if the amplitude of cell size in the population is large. The results are shown in Table 1 and confirm those obtained graphically.

### 3.4. Cell Cycle Regulation and Origin of G2 Phase

**Figure 14:**
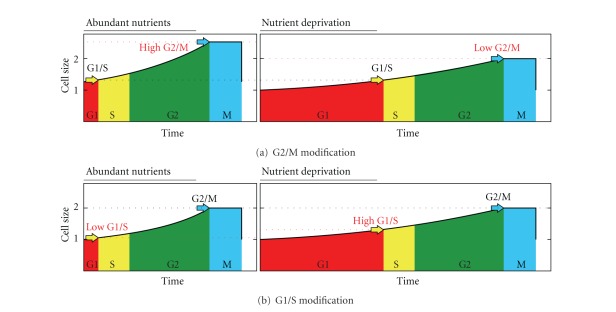
Cell cycle regulation by means of G1 duration: short G1 in rich environments (left) and long in poor environments (right) caused by G2/M modification (a) and G1/S modification (b). G2/M modification implies larger cell sizes in rich environments, whereas modification in G1/S does not.

As the sexual cycle is an advantage in poor environments and the asexual cycle in rich ones, the best strategy would be to choose the best cycle for each condition (although it is likely that environmental richness is inversely related to the probability of cell encounters). It is a fact that facultative sexuality occurs in some present species, for example, *Saccharomyces cerevisiae* [132] and that the ancestor of eukaryotes was likely to be facultative-sexual [147]. A method of achieving this two-fold strategy would be to reduce the G1 phase as much as possible in rich environments. This can be done by modifying the checkpoints, by either reducing G1/S or increasing G2/M enough to force G1/S activation immediately after division (Figure 14).

**Figure 15:**
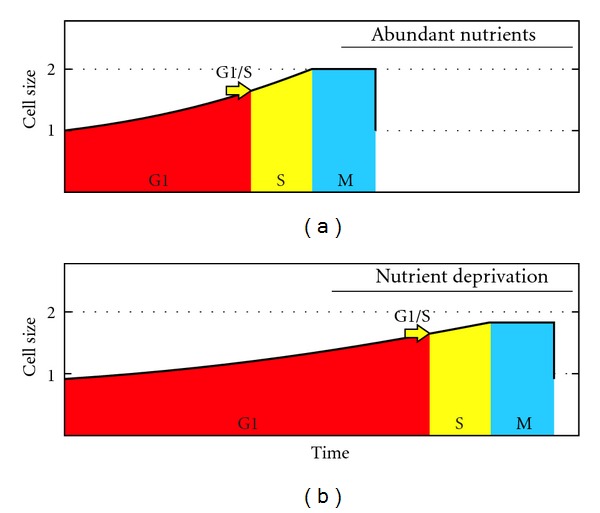
Hypothetical ancestral cell cycles without G2 and growth in S phase. In this case, there would be only one checkpoint, G1/S. If this checkpoint and the duration of the S phase are constant—not dependent on environmental richness—cell size would be slightly smaller in poor environments.

G2/M modification implies larger cell sizes in rich environments, whereas modification in G1/S does not. What path was taken in evolution? We have already revealed (Figure 2) that present yeasts choose G2/M modification regardless of their larger size. The reason may be that it would be easier to modify G2/M if it originated later during evolution. In consequence, there would have been a period without G2 in cellular evolution. Indeed, it is theoretically possible to have a cell cycle without G2 [148, 149]; G1 would be even longer in poor environments and S and M would have the same duration (Figure 15). In such a case, there would be only one checkpoint, G1/S. The end of S phase would trigger the onset of M phase. If this checkpoint (G1/S) and the duration of the S phase are constant—not dependent on environmental richness—cell size would be slightly smaller in poor environments. Such a cell cycle would make the sexual cycle advantageous in a broader range of environmental conditions and for a wider range of cell sizes, although no regulation would have been possible to avoid the sexual cycle in rich environments. This poses the question: was sex the origin of G2?

## 4. Discussion

### 4.1. Growth Curves and Species Optimum Size

The first result of our work—the relationship between the cell growth curve and the optimal size of the species in free-living unicellular organisms—is very surprising. If the graphs and math do not lie—and they do not usually tend to—the only cell growth curve which has no effect on the optimal size of the species is the exponential curve. Any deviation from this curve, either above or below, would generate a selective pressure (which would be greater the more it differed from the exponential curve) towards sizes that were larger or smaller than optimal in the previous generation, changing the size of the species from generation to generation, until some other factor or factors halted the change. We clarify, however, that there are infinite exponential curves, with varying degrees of “steepness” (fast growth) or “recumbence” (slow growth).

The results obtained by some authors on the growth of yeast, which indicate that the best fit curve is the bilinear curve, do not alter our results, since the fit of the exponential curve is also excellent (*r* ^2^ = 0.9949) [68, page 4341] (they are statistically identical), and in any case, the two sections of linear growth continue to meet the pattern of larger size, greater growth.

This is not a result which was sought in our study. In early versions of our cellular automaton, we tested different cell growth curves. The results were that the cell size of the entire population varied, within the limits introduced at the beginning of the simulation, if the growth curve was not exponential. A simple mathematical analysis and a good graphic representation (Figure 4) convinced us of the result. None of the works consulted, nor the works aiming to measure cell growth empirically, or to mathematically model the cell cycle, have obtained our result, and we therefore expect a very rapid response on this point.

### 4.2. Short-Term Advantage for Cell Fusion in Poor Environments

The results presented answer the apparently trivial question of how cell fusion would affect cell cycle duration in a population of asexual free-living unicellular organisms. None of these hypotheses on the origin of sex addresses this issue, although some of them assume a fusion of two or more cells. All of them are based on the genetic benefits of sex, which should theoretically be sufficient to offset its costs (the need for gametes find each other and meiosis). Our approach is therefore completely new (and therefore susceptible to numerous criticisms and improvements).

**Figure 16:**
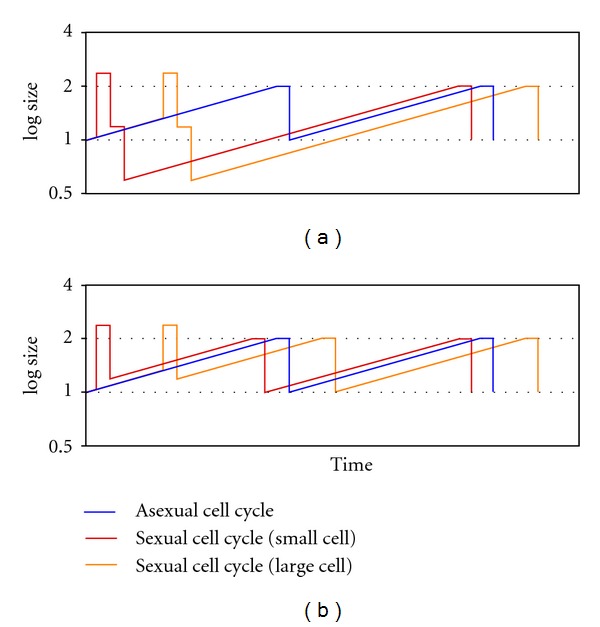
Graphs showing time/size of cell cycles with and without fusion, by assuming that critical size is reached (or exceeded) after cell fusion. (a) with two consecutive divisions. (b) with delayed second division. In this type of cell cycles, the checkpoints of the cell cycle do not depend on the level of ploidy.

Being enthusiasts of evolutionary biology, we were familiar with the cell cycle but not of its complicated (and not yet fully understood) regulation. Therefore, in our first studies on cell fusions, we assumed that, with 1 being the size of the two newly formed daughter cells (by bipartition), and 2 the size that triggers the division, all cell fusions, without exception, would result in a cell size greater than 2, thereby triggering an immediate cell division. Although the cycle would have been considerably shortened, there would still be no multiplication, since two cells fuse to produce another two. But both when the return to the haploid state occurred immediately and when it was delayed, a shortening of the cycle occurred for one of the cells (the smaller) and an extension to the other (the larger) (Figure 16). It became clear that we needed to know more about cell cycle regulation in the present species, and at the same time, to understand the relationship of the phases of the cycle with growth and division, to confirm whether random cell fusions produced a “demographic” advantage.

There is an extensive bibliography on cell cycle regulation, a great deal about the cell growth curve, and almost nothing about the relationship between them. Assuming an exponential growth curve (or approximate) during phases of the cycle in which there is growth, and applying the known relative duration of phases in yeast, we were able to draw a graph as shown in Figure 2(c). Assuming that cycle regulation was similar in the origin of sex (i.e., the critical size in both control points depends on the level of ploidy), the result is that control points are doubled after cell fusion, as a result of duplication in the ploidy level caused by syngamy. Surprisingly, the results are that there was still an advantage for the small cell, and that in the cell cycle the 4n state typical of meiosis is reached naturally. Moreover, by introducing a plateau with no growth, the result is that cell fusion is only advantageous in poor environments.

In the proposed sexual cycle, therefore, the objective of syngamy and meiosis is reproductive. It is not necessary to invoke genetic benefits, although there may be some. This also moves further away from theories that propose an origin of syngamy based on the formation of cysts or syncytia, since they do not in any way justify the expense for duplication of the genome prior to meiosis, which is quite unnecessary in the environmental conditions leading to the formation of such forms of survival. We therefore propose the existence of a long initial period in the evolution of eukaryotes, with alternating rounds of asexual and sexual reproduction, depending on environmental conditions, and where there are no special forms of survival (cysts or spores) or, if there are, which do not involve meiosis. However, the advantages for syngamy that we propose would make it necessary to the previous existence of cellular mechanisms that ensure the return to the haploid state. Possible scenarios would be photosynthetic species with division by multiple fission and those proposed by Cavalier-Smith [25, 47] on cysts and syncytia (without meiosis). Therefore, the evolutionary steps we propose are consistent with the scheme proposed by Maynard Smith and Szathmáry [1]: first a haploid-diploid cycle, with endomitosis and one-step meiosis, followed by a cycle with syngamy and one-step meiosis, and finally a cycle with syngamy and two-step meiosis. The present work provides a previous overlooked potential demographic advantage to the haploid-diploid cycle.

Related to the formation of spores or resting cysts, we believe that there would be an advantage for the syngamy and the ensuing diploid state undiscovered until now. The advantage lies in the lower surface/volume ratio in large cells (diploids by syngamy), because of the smaller investment required to form the spore wall. For instance, when the investment of cell volume to form the haploid spore wall is 25%, this percentage would drop to 20% in the case of a diploid spore. Furthermore, the haploid spores by no means could avoid this disadvantage, because any change in its shape would lead to a greater investment in spore wall. Once restored environmental conditions, spores would be liberated from its envelope and, by a mitotic division without prior S phase, they would return to the haploid state. The result is a kind of haplodiploid cycle that would also explain the existence of a cellular mechanism capable of returning the cell to its former haploid state. Recall that the surface/volume ratio has already been mentioned regarding the hypothesis of “nutrient limitation,” in which haploid cells grow faster than diploids [1, 73]. The main difference between both proposals is that in the “nutrient limitation” any change in cell shape does improve the surface/volume ratio, therefore reducing limitation to growth.

The proposed ancestral sex would be very different from today in several respects. First, the sexual cycle would not be mandatory, as it is for current real gametes, because the cell cycle would complete an asexual cycle in the absence of cell fusion. Second, there would be no mating types (or sexes) in the cells acting as gametes, as they would all be able to be fused together (panmixis). In modern sex, even in unicellular isogamous species, fusion only occurs between gametes of different types. Third, meiosis was achiasmatic (without crossover), and the only possibility of recombination would be chromosomic. Fourth, in poor environments, sexual cycles would be repeated successively until environmental conditions changed. If the environment changed to a rich one, it would return to asexuality. In contrast, if the environment was made even poorer and did not allow any kind of growth, the best option would be the formation of spores or other individual or collective survival forms. Compared with the modern sex of multicellular beings, this aspect of the duration of sex rounds is very different, because in these most of the time (and the number of cell divisions) is asexual.

Would cell fusion have been equally advantageous if cell growth had not followed a roughly exponential curve be contrary to what we hold in this study? Consider first the case that growth was overexponential, a curious situation in which the larger the cell becomes, the more efficient it is. In this case, cell fusion and the subsequent sexual cycle lead the cell to a range of sizes with greater efficiency and it would therefore be possible to shorten the cycle even more. Actually the optimal size of the species would be adjusted to two opposing selective forces, the intrinsic growth curve, which pushes the size to larger sizes; and other factors (environment, predators, physics, etc.) which lead to smaller sizes. Thus the advantages of the sexual cycle would depend on the exact shape of the fitness curve of cell sizes. In the second case, in which the cell growth curve was subexponential (family of curves including linear growth), the cell becomes less efficient the more it grows (perhaps due to limitations imposed by a surface/volume ratio unresolved by the species). The sexual cycle in this case takes its two protagonists to a less efficient range of sizes, and would therefore be less advantageous than in species with exponential growth. It is, however, worth making the same point mentioned above. The optimal size of the species would be a compromise between the selective force to small sizes (derived from the type of growth) and the selective force to large sizes; the advantages of the sexual cycle would depend on the exact shape of the fitness curve of the cell sizes.

Both in graph representations and in computer simulations, we assumed that the cell fusions would only be viable between two cells that are in the G1 phase of the cycle, following the pattern seen today in most eukaryotes [122]. This is a somewhat logical assumption. In G1 the machinery of DNA replication has not yet begun to operate. In the S phase, it has. In G2, the cell size is about to reach—or has already reached—the size of division. In M the complex process of mitosis and cytokinesis is carried out. What seems clear to us is that both cells must be in the same cycle phase. We do not know what mechanisms would be necessary to allow fusion in certain phases of the cycle and to prevent them in others. In budding yeast, the mating factors activate the synthesis of proteins essential for mating, and necessary for cell [150, 151] and nuclear [152] fusion. Thus, the mating pheromone systems act to synchronize the cell cycles of mating partners and to allow the appropriate fusion events [132].

In our hypothesis, sex may or may not have a genetic basis, whereas fusion tendency is somehow hereditary. In the CELLSIMULATOR, it is assumed to be genetic; hence fusion is only inherited by two of four daughter cells when fusions are heterozygotic. In a hypothetical world of organisms without genome, in which information is held in the metabolism itself—as thought by some authors [153, 154] to have occurred in the first period of life on Earth—the sexual advantages shown here would also be valid. If sex appeared in a world of single-cell organisms with genome, sex would have more advantages (not analyzed in this work) relating to hybrid vigor: namely, a higher growth rate and a greater survival rate.

### 4.3. Cell Cycle Regulation and Origin of G2 Phase

Of course, this is the most speculative part of our work, but in view of the undoubted interest of making assumptions about the evolution of the cell cycle, we feel its publication is justified. However, we are aware that by so doing we open our proposals to severe criticism.

The key result of our work, that syngamy can confer an immediate demographic advantage in poor environments, immediately opens the doors to an evolutionary improvement: namely, the regulation by the cells themselves of the type of multiplication, depending on the environmental conditions. In a sexual population in which fusions would be permitted in G1, as soon as environmental conditions changed to becoming more benign, it would be advantageous to avoid fusions. As soon as environmental conditions worsened, it would be advantageous to allow them. Any regulatory mechanism to open or close the door to fusions would be advantageous. If fusions were allowed in G1 and blocked in the remaining phases, by whatever mechanism, there is a simple way (a priori) to display this modulation: by modifying the cell control points so that phase durations shorten or lengthen, as required. Some authors call this facultative alternation [147, 155–158].

Since there are two control points related to size, there would be two possible ways of regulation: by modifying G1/S or modifying G2/M. In the first case, cell size always oscillates around the optimum of the species, since it does not change the control point that triggers cell division. In the second case, the cell size is greater in rich environments. It seems clear that of the two control points G2/M is under greater selective pressure, as this is the point that determines the cell size, a parameter that surely influences its fitness. It therefore seems strange that the current regulation in modern yeast is controlled by G2/M. The evolutionary explanation is the one we like best: that the control point G1/S was more primitive than G2/M and, therefore, less subject to variation. This involves accepting an evolutionary period with a single control point at the end of the G1 phase, a possibility already suggested by Nasmyth [148] and Novak et al. [149] (the fact that the molecular networks regulating the G1/S transition in budding yeast and mammals are strikingly similar in network structure [159]). Indeed, as we have shown in this work, it is theoretically possible to have a cell cycle with G1/S as the only control point related to cell size. In our opinion it is not only possible, but is actually the most logical and simple explanation: it grows until it accumulates sufficient resources, and once it has attained sufficient size and reserves (G1/S), it divides (M and S). However, such a cell cycle would not have been capable of regulating the type of reproduction according to environmental conditions. The proposal that sex was the catalyst for the appearance of the G2 control point and G2/M is tempting.

**Figure 17:**
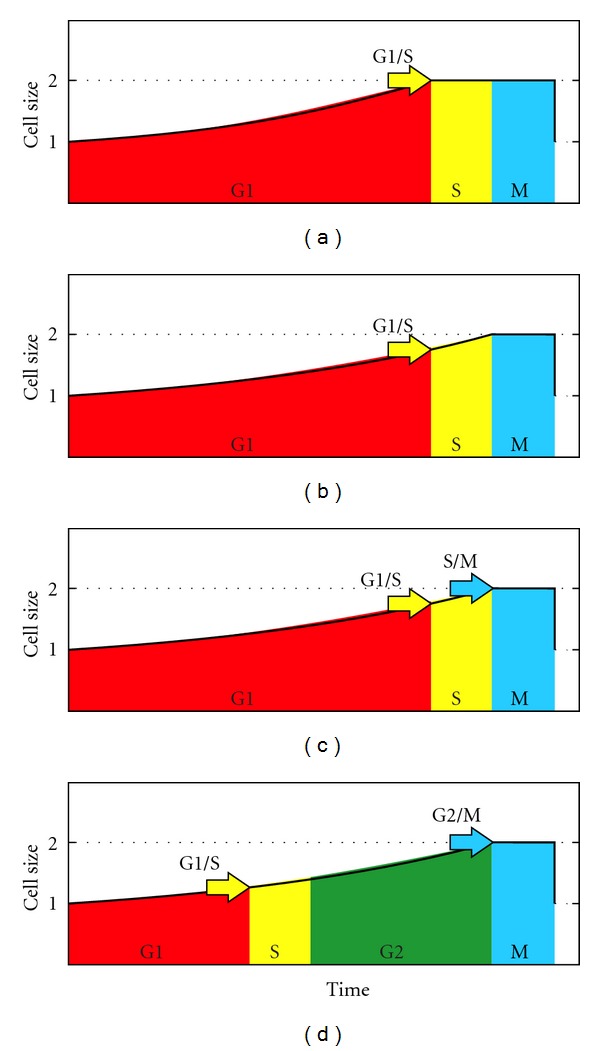
Developmental stages of the eukaryotic cell cycle without the mediation of sex. (a) Primitive stage with phases G1, S, M, a single control point and no growth in the S and M phases. (b) Ditto, with growth in S. The cell size is slightly higher in rich environments than in poor ones. (c) To avoid this problem, a new control point appears, prior to M, which ensures that the cell division size is always optimal. (d) Because of metabolic efficiency, the S phase of the cycle moves to the center, and a new phase, G2, appears.

However, it is also possible to raise another stage of evolution that explains the appearance of G2 unrelated to sex, based on the evolution of the checkpoints and the cycle profile (Figure 17): (1) a primitive stage with G1-S-M phases in which cells stop growing in the S and M phases. There is only one control point at G1/S. In this case the control point is perfectly adjusted to the optimal size of the species. (2) The cells “learn” to grow during the S phase. This implies minor variations in size depending on the environmental conditions, as in rich environments growth in the S phase is higher than in poor ones. (3) Prior to M, a new control point appears which ensures that the cell division size is optimal for the species: G1-S-M cycle with two control points. (4) For reasons of metabolic efficiency, the S phase is “pushed” to the center of the cycle, thus the genome/cytoplasm relation remains near the optimum for longer. Bear in mind that the relationship between nuclear and cytoplasmic volume [113, 160–162] seems fairly well established [40, 73, 110, 163–166]. The fact that in fission yeast the increased growth rate in the second stage of the cycle depends on the synthesis of DNA [68, page 4343] also points in the same direction. Therefore, a cell growth phase appears after S, that is, G2. The second control point would not have changed, but would now be called G2/M.

### 4.4. A New Approach

Time is a key factor in our hypothesis on the advantages of syngamy. Probably, the main difference with other hypotheses is the use of generation time—along with death rate—as a comparison method between strategies. All preceding hypotheses on the origin or maintenance of sex involve no time unit at all; instead they use survival probabilities related to individual fitness. These hypotheses assume that generation (doubling time in unicellular species) is the same for all individuals, as derived by the iconography used in them. The second main difference is implied in the concept of *generation time* itself: one of the goals of single-cell organisms is to reduce duplication time, and hence to increase their birth rate. That is, the *quantity* of living matter is of great importance. All the organisms in a population compete for resources (matter and energy) and transform them into living matter. Indeed, the cell cycle is shorter in rich environments. Natural selection favors cells with a shorter cycle, as they are able to duplicate more times within the same period.

## Supplementary Material

CELLSIMULATOR: Software (Excel worksheet) to simulate the biological evolution of a single-cell population and to test the hypothesis regarding the advantages of syngamy.
VIDEO: Animated graph showing a population dynamics in which the sexual strategy spreads throughout a previously asexual population (X-axis shows cell ages and Y-axis cell sizes). Cell cycle phases are shown in different colors.

## Figures and Tables

**Figure 18:**
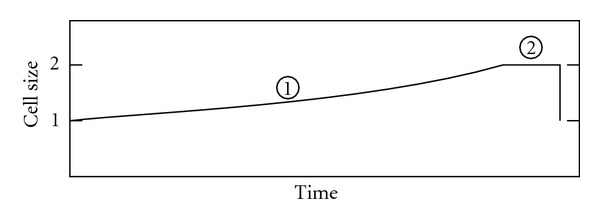
Asexual cell cycle.

**Figure 19:**
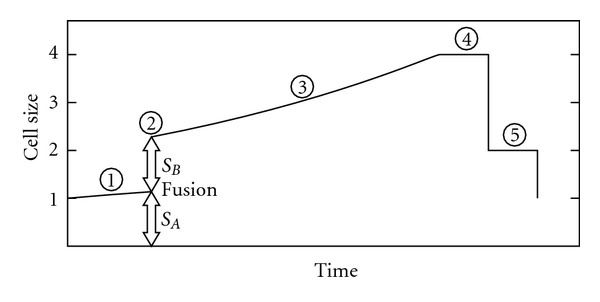
Sexual cell cycle.

**Figure 20:**
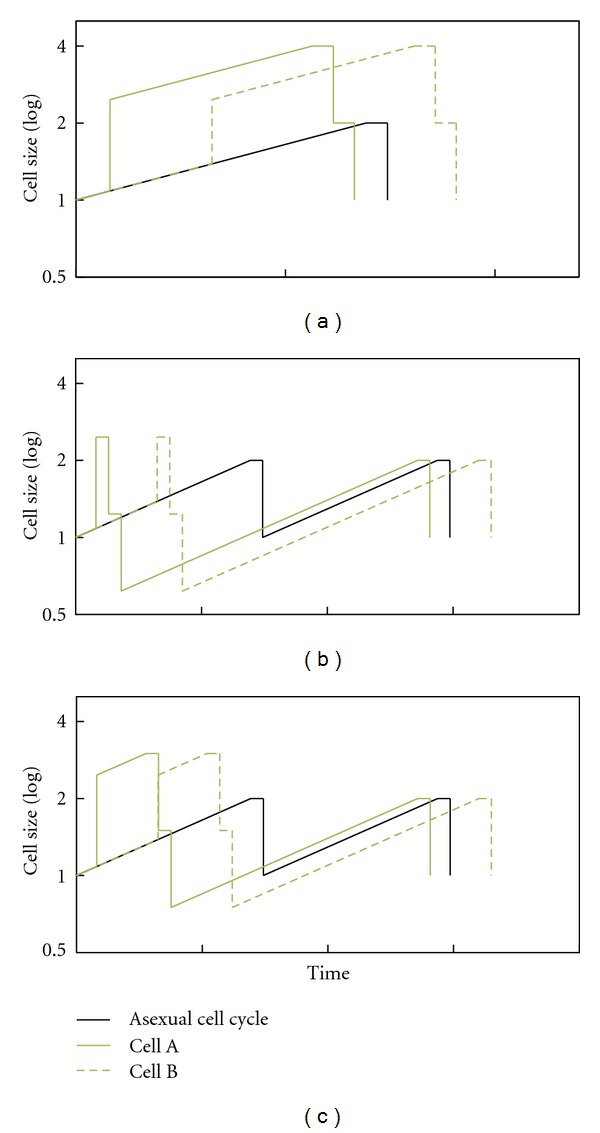
Comparison of asexual and sexual cell cycles with different values of the G2/M checkpoint. (a) 4 (value used in the main text). (b) 2. (c) 3. Graphs are semilogarithmic.

**Figure 21:**
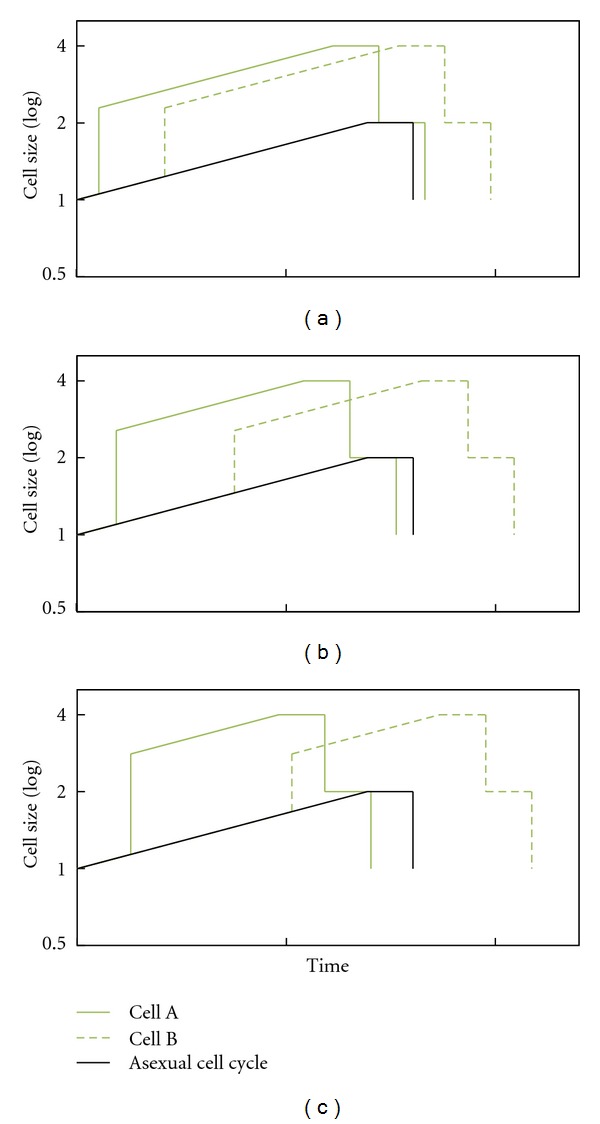
Comparison of asexual and sexual cell cycles with different values of the G1/S checkpoint. (a) 1.3. (b) 1.6. (c) 1.9. Graphs are semilogarithmic.

**Figure 22:**
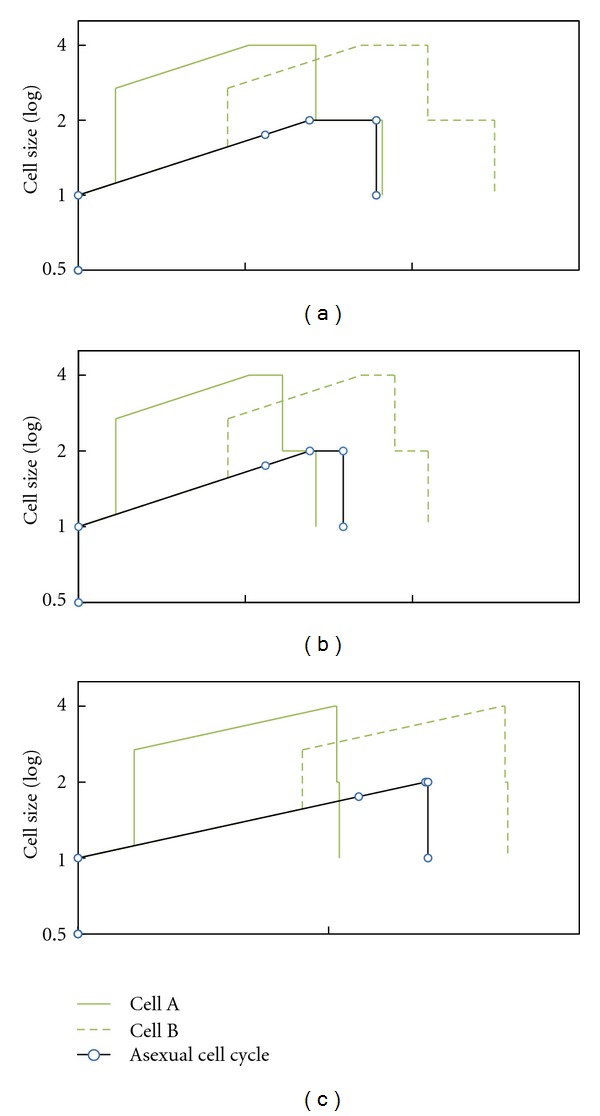
Comparison of asexual and sexual cell cycles with different plateau duration. (a) Plateau = 200. There is no advantage for the sexual cell cycle. (b) Plateau = 100. There is a slight advantage for the smaller cell in the sexual cell cycle. (c) Plateau = 5. There is a great advantage for the smaller cell. Graphs are semilogarithmic.

**Table 1:** Ratio of the duration of the sexual cycle of the small cell and the asexual cycle, expressed as a percentage, depending on environmental conditions (*k*) and the relationship between the sizes of the two cells that fuse (*p*). Percentage below 100% indicates reduced duration of cell cycle and therefore advantage to syngamy. The rows below show the asexual cycle duration (arbitrary units) and the percentage of the cycle without growth (the plateau end of the cycle).

		*k* (growth rate)					
		0.001	0.002	0.004	0.008	0.016	0.032
*p*	1	103%	105%	110%	119%	132%	148%
	1.2	89%	92%	98%	108%	122%	141%
	1.4	77%	81%	87%	97%	114%	134%
	1.6	66%	70%	76%	88%	106%	128%
	1.8	56%	60%	67%	79%	98%	123%
	2	46%	50%	58%	71%	92%	118%

Asexual cell cycle duration		713	367	193	107	63	42
Plateau percentage		3%	5%	10%	19%	32%	48%

**Table:** (a)

*i*, *j *		1	2	3	4	5	6	7	8	9	10
	*N*	10.00	8.82	7.78	6.86	6.05	5.34	4.71	4.15	3.66	3.23
1	10.00	100.00	88.20	77.79	68.61	60.52	53.38	47.08	41.52	36.62	32.30
2	8.82		77.79	68.61	60.52	53.38	47.08	41.52	36.62	32.30	28.49
3	7.78			60.52	53.38	47.08	41.52	36.62	32.30	28.49	25.13
4	6.86				47.08	41.52	36.62	32.30	28.49	25.13	22.16
5	6.05					36.62	32.30	28.49	25.13	22.16	19.55
6	5.34						28.49	25.13	22.16	19.55	17.24
7	4.71							22.16	19.55	17.24	15.21
8	4.15								17.24	15.21	13.41
9	3.66									13.41	11.83
10	3.23										10.43

**Table:** (b)

*i*, *j *		1	2	3	4	5	6	7	8	9	10
	*N*	10.00	8.82	7.78	6.86	6.05	5.34	4.71	4.15	3.66	3.23
1	10.00	4.89%	4.32%	3.81%	3.36%	2.96%	2.61%	2.30%	2.03%	1.79%	1.58%
2	8.82		3.81%	3.36%	2.96%	2.61%	2.30%	2.03%	1.79%	1.58%	1.39%
3	7.78			2.96%	2.61%	2.30%	2.03%	1.79%	1.58%	1.39%	1.23%
4	6.86				2.30%	2.03%	1.79%	1.58%	1.39%	1.23%	1.08%
5	6.05					1.79%	1.58%	1.39%	1.23%	1.08%	0.96%
6	5.34						1.39%	1.23%	1.08%	0.96%	0.84%
7	4.71							1.08%	0.96%	0.84%	0.74%
8	4.15								0.84%	0.74%	0.66%
9	3.66									0.66%	0.58%
10	3.23										0.51%

**Table:** (c)

35%	No fusion probability	
65%	Fusion probability	
20%	Fusions between cells of the same size	
80%	Fusions between cells of different size	

		%

Difference between age classes	1	17%
	2	15%
	3	13%
	4	10%
	5	8%
	6	7%
	7	5%
	8	3%
	9	2%

**Table 3:** Percentage of anisogamous fusions (between different age classes) according to the instantaneous death rate and the instantaneous probability of fusion in a discrete analysis with ten age classes. The higher the percentage of anisogamous fusions, the more advantages for syngamy.

Probability of “anisogamous” fusions (10 age classes)									
		Death rate							
		0	0.001	0.01	0.1	0.2	0.4	0.6	0.8
Fusion probability	0	82%	82%	82%	80%	76%	60%	40%	20%
	0.001	82%	82%	82%	80%	76%	60%	40%	20%
	0.01	82%	82%	82%	80%	75%	59%	40%	20%
	0.1	80%	80%	80%	76%	70%	54%	36%	18%
	0.2	76%	76%	75%	70%	63%	48%	32%	16%
	0.4	60%	60%	59%	54%	48%	36%	24%	12%
	0.6	40%	40%	40%	36%	32%	24%	16%	8%
	0.8	20%	20%	20%	18%	16%	12%	8%	4%
	1	0%	0%	0%	0%	0%	0%	0%	0%

## Appendices

### A. Cell Cycle Duration

#### A.1. Asexual Cell Cycle Duration

We can divide the cell cycle in two sections (Figure 18): in the first one (1), the cell grows in an exponential curve. In the second section (2), the cell stops growing (the final plateau).

The exponential growth curve is *S*_*t*_ = *S*_0_ · *e* ^*kt*^, where *S*_*t*_ is the cell size in time *t*, *S*_0_ is the initial cell size, *k* is the growth rate (dependent on environmental richness), and *t* is the time. The first section ends when the cell size has doubled, that is, *S*_*t*_ = 2 · *S*_0_. Introducing this expression into the exponential growth curve and solving it, *T*_1_ = (ln⁡⁡(2))/*k*.

Let us call *M* to the duration of the second section: *T*_2_ = *T*_*M*_.

The total time needed to complete a cell cycle is the sum of both times:

(A.1) $$T=\frac{\ln\left(2\right)}{k}+T_{M}.$$

#### A.2. Sexual Cell Cycle Duration

This cycle includes 5 sections: (1) the cell grows in an exponential curve; (2) the cell fuses; (3) the cell continues to grow in an exponential curve; (4) the cell stops growing and performs the first division; (5) the cell performs the second division (Figure 19) as follows:

1. growth curve in the first section is exponential: *T*_1_ = ln⁡(*S*_*A*_/*S*_0_)/*k*,
2. let us assume fusion is instantaneous: *T*_2_ = 0,
3. the new cell keeps on growing. This section ends when cell size is four times the initial size: *T*_3_ = ln⁡⁡(4*S*_0_/(*S*_*A*_ + *S*_*B*_))/*k*.

Let us call *M* the duration of the two last periods:

(4) *T*_4_ = *T*_*M*1_, and
(5) *T*_5_ = *T*_*M*2_.

The total time needed to complete a cycle is the sum of all section times:

(A.2) $$T=\frac{\ln\left({4S}_{A}/\left(S_{A}+S_{B}\right)\right)}{k}+T_{M1}+T_{M2}.$$

Case 1 Both cells have the same size when fusion occurs, *S*_*A*_ = *S*_*B*_. Now we solve (A.2):

(A.3) $$\;T=\frac{\ln\left(2\right)}{k}+T_{M1}+T_{M2}.$$

Compare this result with the asexual duration (A.1):

(A.4) $$T_{\text{sexual}}-T_{\text{asexual}}=T_{M2}.$$

The result is that the duration of the sexual cycle is longer than the asexual due to the time required for the second division.

Case 2 Cells are different sizes, *S*_*B*_ = *p* · *S*_*A*_:

(A.5) $$T=\frac{\ln\left(4/\left(1+p\right)\right)}{k}+T_{M1}+T_{M2}$$

with *p* values less than 1; we obtain the cell cycle duration of the largest cell, whereas with values between 1 and 2, we obtain the duration of the small one. Introducing different values of *k* and *p* in a double math table we can calculate the exact reduction or enlargement of the small cell cycle. The results are shown in Table 1.

### B. Statistical Analysis of Cell Sizes in Fusion

For the sexual cycle to be shorter than the asexual cycle, the fusions must only occur between cells of different sizes, because if not, as we have seen, the cell cycle is longer than the asexual cycle. It is therefore necessary to clarify whether most fusions occur between cells of similar or different sizes.

We will analyze this by making a discrete analysis, which is the easiest. We divide the duration of the G1 phase in equal intervals of time (ten classes in our analysis) (Table 2). Every individual has a probability of dying *d* and fuse *f*, which we will assume remains constant throughout the cycle (*d* = 2%, *f* = 10% in our analysis). If an age class has *n*_*i*_ haploid individuals (suitable for fusion), the next will have

(B.1) $$n_{i+1}=n_{i}\cdot \left(1-d\right)\cdot \left(1-f\right).$$

The cumulative result of this process, means a distribution of ages/sizes with a negative exponential distribution for the population of candidates to fuse. Introducing the distribution of ages/sizes in a double-entry math table, we first calculate the number and then the percentage of each possible fusion partner. The result of this analysis is that 20% of fusions occur between cells of the same age class (and therefore similar size), and 80% among different classes. Furthermore, this group is distributed asymmetrically, with a high probability of fusions between slightly different-sized cells and little chance of fusion between very different ones.

Using the same table with ten age classes we can analyze the influence of the death rate and the probability of fusion in the percentage of fusions between different age classes (Table 3).

### C. Modeling of the Conditions Which Allow the Introduction of Sex: Relative Duration of the G1 Phase, Relative Duration of the Plateau, and Probability of Cellular Fusion

We have developed a model (with a spreadsheet) which allows us to compare the demographic growth rates of sexual and asexual populations at any intermediate time during the transition of a population from an asexual to a sexual state. The mathematical basis used is explained below.

The size of the population varies over time based on the rate of growth, according to the widely-known expression:

(C.1) $$\;N_{t}=N_{0}e^{rt},$$

where *N* is the population size at time *t*, and *N*_0_ the initial population.

In demographics, the growth rate of a population (*r*) is determined by birth rate (*b*), death rate (*d*), immigration (*i*), and emigration (*e*), by means of the simple expression:

(C.2) r=b−d+i−e.

A population is in equilibrium (the total number of individuals neither increases nor decreases) when the rate of growth is zero. Our theoretical model does not include immigration or emigration, so that

(C.3)  r=b−d.

We have considered the death rate to be constant, that is, independent of the age or size of the individual. Moreover, as this is a case of single-cell organisms, the birth rate occurs solely and exactly at the end of the cell cycle, thereby generating a new cell in one generation time, so that

(C.4) $$\frac{\Delta N\left(T\right)}{N}=e^{bT}=2,$$

and isolated thus

(C.5) $$b=\frac{\ln\left(2\right)}{T}.$$

Applying this result to the formula for the population size, we obtain

(C.6) $$N_{t}=N_{0}e^{((\ln\left(2\right)/T)-d)t}.$$

However, the result thus obtained is only exact when the time is a whole number of generation times, or when the population is in equilibrium. In all other cases the age pyramid generates deviations in the real growth rate, due to variance in the generation time between the cells and to random deviations in the death rate. As a result, this equation offers only an *average* forecast of how population size will evolve.

We have modeled the introduction of sex in asexual populations in equilibrium, that is, with a zero growth rate, and therefore

(C.7) $$\;r=0,\;\;b=d,\;\;T_{e}=\;\frac{\ln\left(2\right)}{d},\;\;k=\;\frac{\ln\left(2\right)}{T_{e}-T_{M}},$$

where *T*_*e*_ is the time of asexual generation (in equilibrium), *k* is the rate of cell growth for which the duration of a cell cycle is exactly *T*_*e*_, and *T*_*M*_ is the duration of the plateau (see (A.1)).

In a population in which there are sexual cells, the generation time will depend—if there is cell fusion—on the size of both contenders. To obtain the growth rate of each subpopulation based on its sizes (when fusion occurs), we use the equation for generation time obtained in Appendix A:

(C.8) $$r_{AB}=\frac{\ln\left(2\right)}{\left(\ln\left(4/\left(1+S_{A}/S_{B}\right)\right)/\text{k}\right)+2T_{M}}-d,$$

where *A* and *B* are the cells which fuse, and *S*_*A*_ and *S*_*B*_ are their respective sizes at the time of fusion.

Now we need to calculate the percentage for each size combination involved in the fusions. To do so we create a double-entry table in which the number of individuals in each age class decreases exponentially with the death rate *and* with the probability of fusion. The final growth rate of the population is obtained from a weighted average which takes into account the proportion of cells in the population—a function of the number of cells in each age class and generation time—which undergoes each cycle:

(C.9) $$\frac{N_{AB}}{N}=\frac{N_{A}N_{B}\left(e^{-dT_{AB}}-1\right)}{\sum_{i,j}N_{i}N_{j}\left(e^{-dT_{ij}}-1\right)},$$

where *i* and *j* are the age classes, and

(C.10) $$\;r=\ln\left(\underset{i,j}{\sum }\frac{N_{i}N_{j}\left(e^{-dT_{ij}}-1\right)}{\sum_{i,j}N_{i}N_{j}\left(e^{-dT_{ij}}-1\right)}e^{((\ln\left(2\right)/T_{ij})-d)t}\right).$$

Using (C.10), we calculated growth rates of sexual and asexual subpopulations for some combinations of G1 duration, plateau duration and fusion probabilities. When sexual growth rate is bigger than asexual one, sex would be able to thrive.

The results are shown in Figure 12 (3D graph). Modeled growth rate is depicted using the brown surface. This surface shows fusion probability threshold, volume under the surface means a bigger sexual growth rate, hence, favourable conditions for sex to thrive. The CELLSIMULATOR simulations in which sex succeeded are highlighted in green and in red where it did not. The agreement between the simulations and the model is excellent. It should be noted that the probability of fusion has much less influence on the advantage of sex than the duration of G1 and the plateau (the transition of the conditions which permit the introduction or not of sex occurs abruptly, as can be seen by the very steep slope in the graph).

### D. Influence of Sexual G2/M Checkpoint on Sexual Cell Cycle Duration

In the presentation of this hypothesis, we assumed that sexual cells by syngamy would show a doubled critical size. Here we analyze various different cases. The graphs below (Figure 20) are semilogarithmic to make them easier to understand.

In these cases, cell A is located at 20% of G1 and cell B at 80%. G1/S critical size is 1.5. In Figure 20(a), we again show the cycles when diploid G2/M is double haploid, that is, 4. In the Figure 20(b), we analyze the case in which G2/M is not modified by ploidy, as it is the same as haploid, that is, 2. Division begins immediately after fusion. This cycle, apparently shorter for both cells, yields four tiny cells. If these cells were viable, their growing time phase would be extra-long until they reached the critical size again. Once two cell cycles are depicted, only the cell A cycle is shorter than the asexual cycle. Such a strategy would be astonishingly advantageous if, after the first cycle tiny sexual cell A fuses with another asexual cell, although this huge advantage would only last during the transformation of the population from asexual to sexual. Figure 20(c) shows an analysis of the case with G2/M = 3. The resulting cycles again show a sexual advantage after the second cycle.

In consequence, sex would be an advantageous strategy whatever the behavior of the checkpoints in the diploid cells is.

### E. Influence of Asexual G1/S Checkpoint on Sexual Cell Cycle Duration

The relative duration of asexual G1 is a key factor in quantifying sexual cell cycle advantage; the longer the G1, the greater the range of sizes when fusion occurs. We analyze three cases involving a sexual cell A at 20% of G1 and a cell B at 80% of G1, where G1/S is 1.3, 1.6, and 1.9, respectively, (Figure 21).

### F. Influence of Plateau Duration on Sexual Cell Cycle Duration

The relative duration of the plateau without growth is also a key factor in quantifying sexual cell cycle advantage. We analyze three cases involving a sexual cell A at 20% of G1 and a cell B at 80% of G1, being this phase the 75% of the cell cycle, with different duration of the plateau (Figure 22). As shown in Appendix A, there is a time length of the plateau after which the sexual cell cycle is no longer shorter for the smaller cell (Figure 22(a)).
